# Novel Methods of Automated Quantification of Gap Junction Distribution and Interstitial Collagen Quantity from Animal and Human Atrial Tissue Sections

**DOI:** 10.1371/journal.pone.0104357

**Published:** 2014-08-08

**Authors:** Jiajie Yan, Justin K. Thomson, Xiaomin Wu, Weiwei Zhao, Andrew E. Pollard, Xun Ai

**Affiliations:** 1 Department of Cell and Molecular Physiology, Loyola University Chicago, Maywood, Illinois, United States of America; 2 Department of Biomedical Engineering, University of Alabama at Birmingham, Birmingham, Alabama, United States of America; University of Minnesota, United States of America

## Abstract

**Background:**

Gap junctions (GJs) are the principal membrane structures that conduct electrical impulses between cardiac myocytes while interstitial collagen (IC) can physically separate adjacent myocytes and limit cell-cell communication. Emerging evidence suggests that both GJ and interstitial structural remodeling are linked to cardiac arrhythmia development. However, automated quantitative identification of GJ distribution and IC deposition from microscopic histological images has proven to be challenging. Such quantification is required to improve the understanding of functional consequences of GJ and structural remodeling in cardiac electrophysiology studies.

**Methods and Results:**

Separate approaches were employed for GJ and IC identification in images from histologically stained tissue sections obtained from rabbit and human atria. For GJ identification, we recognized N-Cadherin (N-Cad) as part of the gap junction connexin 43 (Cx43) molecular complex. Because N-Cad anchors Cx43 on intercalated discs (ID) to form functional GJ channels on cell membranes, we computationally dilated N-Cad pixels to create N-Cad units that covered all ID-associated Cx43 pixels on Cx43/N-Cad double immunostained confocal images. This approach allowed segmentation between ID-associated and non-ID-associated Cx43. Additionally, use of N-Cad as a unique internal reference with Z-stack layer-by-layer confocal images potentially limits sample processing related artifacts in Cx43 quantification. For IC quantification, color map thresholding of Masson's Trichrome blue stained sections allowed straightforward and automated segmentation of collagen from non-collagen pixels. Our results strongly demonstrate that the two novel image-processing approaches can minimize potential overestimation or underestimation of gap junction and structural remodeling in healthy and pathological hearts. The results of using the two novel methods will significantly improve our understanding of the molecular and structural remodeling associated functional changes in cardiac arrhythmia development in aged and diseased hearts.

## Introduction

Cardiac arrhythmias are associated with a significantly diminished quality of life and high mortality [1], [2]. Because gap junctions (GJs) are the primary membrane structures responsible for conducting cardiac impulses between adjacent cardiac myocytes, and increased interstitial collagen (IC) tends to physically separate myocytes from one another [3]–[7], quantifying both GJ and IC as a component of histologic reconstruction is important for improved descriptions of structural remodeling. Accumulated evidence suggests that reduced GJs and increased IC lead to slowing of cardiac action potential propagation and increase propensity for cardiac arrhythmias [8]–[16]. However, quantifying GJ and IC in automated and systematic ways has proven to be challenging.

Cardiac muscle fibers are circumferentially and longitudinally arranged in three-dimensional space; therefore, the abundance and distribution of GJs vary at different imaging focal planes. In healthy ventricular myocardium, this issue is potentially manageable because of the organized muscle structure. The more pronounced structural heterogeneity of healthy atrial myocardium and pathologically enhanced heterogeneity in diseased myocardium poses a risk that use of quantified fluorescent signals could either underestimate or overestimate the amount of GJ proteins. Thus, this risk potentially exists in imaging quantification for both diseased and normal hearts. N-Cadherin (N-Cad) is an important component of intercalated discs (IDs) that are critical for anchoring connexins (e.g. connexin43 (Cx43)) to form functional GJ channels at the ID between adjacent myocytes [3], [17]–[19]. Here, we have developed a novel quantitative method to segment ID-associated and non-ID-associated GJs since we and others have previously demonstrated an unchanged expression of N-Cad in failing and aged hearts [16], [20], [21]. Our main innovation here involved development of a quantitative blur algorithm that used N-Cad as an internal reference to quantify both distribution and abundance of Cx43 from Cx43/N-Cad double immuno-stained cardiac tissue sections.

Similarly, quantitative assessment of IC is critical in understanding the role of structural remodeling in arrhythmia development in addition to GJ remodeling [18], [22]–[25]. However, the current approach for such assessment involves manually outlining collagen areas from Masson's Trichrome (MT) stained tissue. Automated computational approaches are largely unavailable. Here, we are the first to develop and describe a computer-based algorithm using a thresholding approach that quantifies IC in images obtained from MT stained atria.

## Methods and Results

### 1. Specimen preparation

This investigation conforms to the Guide for the Care and Use of Laboratory Animals (NIH Publication, 8th Edition, 2011) and was approved by the Loyola University Chicago and University of Alabama at Birmingham Institutional Animal Care & Use Committees. Four young New Zealand White rabbits were injected with one dose of ketamine (44 mg/kg I.M.) followed by 5% isoflurane delivered in 100% oxygen to induce a surgical plane of anesthesia prior to cardiac excision. Left atria (LA) were dissected, then either flash frozen in liquid nitrogen for immuno-blotting or 24 hr fixation with 10% formalin for immunohistochemistry studies. LA tissue from 18 human donor hearts (which could not be used for heart transplantation but with normal cardiac function) was obtained from Illinois Gift of Hope Organ & Tissue Donor Network (GOH) and Alabama Organ Center (AOC). Table 1 shows unidentified general data (age, sex, race) of the donors. The studies were approved by the Human Studies Committees of Loyola University Chicago, Illinois GOH, University of Alabama at Birmingham, and AOC. All data are presented as Mean ± SEM. Differences between multiple groups or any two groups were evaluated using one-way ANOVA (Post-hoc Tukey test) and student t-test. A p<0.05 was considered to be significant.

**Table 1:** Unidentified general data of the donors.

Age (yr)	Sex	Race
19	F	CAU
34	F	CAU
61	M	CAU
78	F	AA
82	F	CAU
34	M	AA
68	F	CAU
61	M	CAU
26	F	CAU
19	F	CAU
47	F	CAU
52	F	CAU
53	F	CAU
58	F	AA
26	M	CAU
29	M	CAU
52	M	CAU
68	M	CAU

F, Female; M, Male; CAU, Caucasian; AA, Africa American.

### 2. Immunohistochemistry

Rabbit and human atrial tissue preparations were formalin-fixed, embedded and sectioned using a standard procedure. Paraffin-embedded LA sections were de-waxed with xylene and hydrated through graded ethanol solutions followed by a standard microwave antigen retrieval procedure. Double Cx43 and N-Cad immunohistochemistry (IHC) staining was performed using fluorescence labeling techniques as previously described [16], [26]. In brief, tissue sections were placed in 10% normal goat or donkey serum. The slides with tissue sections were then incubated with specific primary antibodies including Cx43-T (Abcam) and N-Cad (Zymed) and fluorescent conjugated secondary antibodies (Molecular Probe). Nuclei were stained with DAPI contained in a mounting medium.

### 3. Confocal image acquisition

**Figure 1:**
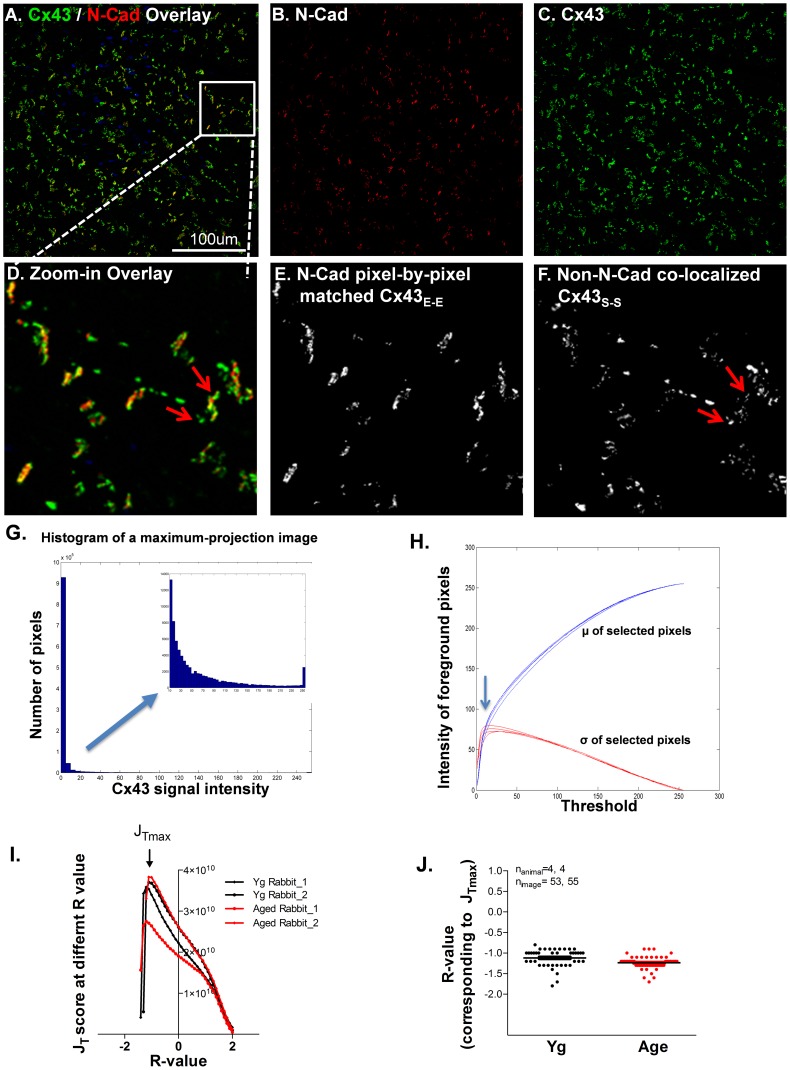
Confocal images of double immunofluorescence staining with Cx43 (green) and N-Cadherin (N-Cad; red) antibodies in rabbit left atrium (LA). **A-C**. Representative confocal images of Cx43/N-Cad overlay (**A**), N-Cad (red; **B**) and Cx43 (green; **C**). **D-F**. Enlarged images from a cropped area of image A including the overlay image (**D**), N-Cad pixel-by-pixel matched Cx43 (**E**), and N-Cad non-pixel-by-pixel Cx43 (**F**). Arrows indicate stellate Cx43 pixels that are in close proximity with the N-Cad pixel but do not co-localize with N-Cad pixel-by-pixel. **G**. Representative histogram of quantified pixel intensity of Cx43 stained image. **H**. Representative plot for the mean intensity (blue curve) and standard deviation (red curve) of foreground pixels at different thresholds. **I**. Representative plot of J_T_ scores corresponding to R-value of four images from two young and two aged rabbit LA. **J**. Data plot of maximum J_T_ scores of the images from four young and four aged rabbit LA.

Images of Cx43 and N-Cad double immuno-stained atrial tissue sections were obtained using a Z-stack mode with a laser scanning confocal microscope (Zeiss; 40x magnification). Twenty to twenty five Z-stack confocal images with muscle fiber orientation parallel to the focal plane from each specimen were acquired with each Z-stack image file containing sequential images from 8–10 scanning layers. Each individual image of the Z-stack file was converted into 6–10 “tif” files at 8-bit data depth. A maximal projection image combining all the sequential images was also processed and saved using “tif” format. Each confocal fluorescent image “tif” file was encoded on three channels: red stained N-Cad channel (R), green stained Cx43 channel (G), and blue stained nuclei channel (B). Two grayscale images were converted from the R and G channels for quantitative image processing (Figs. 1A-1C) such that pixels with value 0 represented complete negative staining and pixels with value 255 represented complete saturation (Fig. 1G). According to a threshold method developed by Ostu N, [27] an ideal threshold that differentiates the foreground from the background and gives the minimized weighted sum of within-class variances can be determined by a maximal value of the J_T_ score where J_T_ = [P_1_*P_2_*(μ_1_-μ_2_)^2^]/(σ_1_^2^+σ_2_^2^). P_1_ and P_2_ are the number of pixels in the two groups (foreground and background), μ_1_ and μ_2_ are the mean intensity level of the two groups, and σ_1_ and σ_2_ are their standard deviations. For each image, we found that mean intensity of selected foreground pixels was increased along with incrementally changed thresholds, meanwhile the value of the standard deviation was initially increased and then decreased after a maximal peak was reached (Fig. 1H). Thus, the initial threshold for positively stained Cx43 pixels was selected based on the maximal standard deviation (σ_green_) on each image (Fig. 1H). A simplified formula: µ_green_+R* σ_green_ was then applied in our algorithm for each individual image to define an optimal threshold (where R is a variable that gives a maximal J_T_ score and µ_green_ is the mean of selected Cx43 pixels at the initial threshold). To determine optimal Rs for Cx43 immunostained images from young and aged rabbit atrial sections (n = 4, 4), J_T_ values of a total of 108 images with Rs ranging from −3 to 3 were calculated. We found that the maximum J_T_ values of the images from both young and aged rabbit atrial sections were very close to an R-value of −1.18 (μ_Yg_ = −1.12, σ_Yg_ = 0.027, µ_Aged_ = −1.24, σ_Aged_ = 0.021; Figs. 1I & 1J). Positively stained Cx43 pixels were identified based on the optimal R-value. Finally, the sum of intensities of all positively stained pixels from each image was calculated as the quantified result.

### 4. Quantitatively determining Cx43 distribution

While most Cx43 pixels co-localized with N-Cad pixels end-to-end at two adjacent myocytes, some Cx43 pixels did not co-localize with N-Cad and instead were distributed to a lateral border between adjacent myocytes. Based on these natural features of Cx43, two categories of Cx43 pixels were defined in our quantitative analyses: N-Cad co-localized end-to-end Cx43 (Cx43_E-E_; Fig. 1E) and non- N-Cad associated laterally and distributed side-by-side Cx43 (Cx43_S-S_; Fig. 1F).

#### 4.1. Determining the relationship between Cx43 and N-Cad using a pixel-by-pixel quantitative algorithm and biochemical co-immunoprecipitation assay

**Figure 2:**
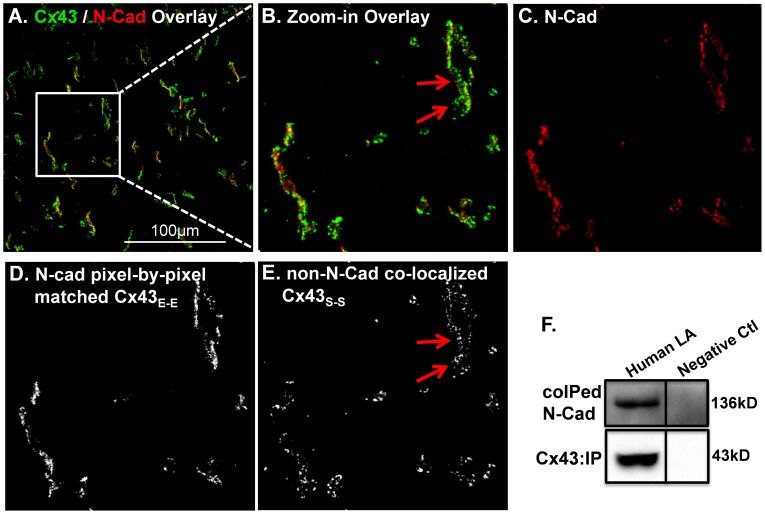
Interaction between Cx43 and N-Cad proteins in human LA. **A**. Representative double immunostaining image of Cx43 (green) and N-Cad (red). **B-E**. Enlarged images from a cropped area of image A including the Cx43/N-Cad overlay image (**B**), N-Cad (red; **C**), N-Cad pixel-by-pixel matched Cx43 signals (**D**), and non-N-Cad co-localized Cx43 (**E**). Arrows indicate stellate Cx43 pixels are in close proximity with the N-Cad pixel but do not pixel-by-pixel co-localize with N-Cad. **F**. Immunoblotting images of co-immunoprecipitated N-Cad protein with immunoprecipitated Cx43 proteins. Right column is the immunoprecipitated negative control without primary Cx43 antibody.

Initially, we used a pixel-by-pixel determination approach to define the relationship between double immuno-stained Cx43 and N-Cad fluorescent signals on atrial tissue sections (Fig. 1). Pixels were considered as N-Cad co-localized Cx43 (Fig. 1E) if they were positively stained pixels in both Cx43 and N-Cad channels. Any positively stained Cx43 pixels that were negatively stained in the N-Cad channel were considered as N-Cad non-colocalized Cx43 pixels (Fig. 1F). Integrated intensity of all positively stained Cx43 pixels was defined as total Cx43 (Cx43_T_). However, we discovered that ID associated Cx43 did not always co-localize with N-Cad in a pixel-by-pixel fashion in the rabbit LA tissue sections (Figs. 1E & 1F). Figs.1D-1F shows a typical example of Cx43 clusters that distributed closely with the N-Cad plaques in general, but some Cx43 pixels (red arrows of Figs. 1D,1F) did not overlap with the N-Cad pixels, despite being in close proximity. Moreover, this phenomenon found in rabbit atrium was also discovered in human atrial tissues (Figs. 2A-2E). Since we were concerned that such pixel-by-pixel determination of N-Cad matched Cx43 could lead to an underestimation of Cx43_E-E_ and overestimation of Cx43_S-S_, we next sought to consider whether stellate Cx43 signals were free-standing Cx43 proteins or were closely associated with N-Cad on IDs. To define the relationship between Cx43 and N-Cad, we performed immunoprecipitation experiments using human atrial tissue homogenates. Cx43 proteins were immunoprecipitated (IPed) with a specific anti-Cx43 antibody. We then performed immunoblotting to detect co-immunoprecipitated (co-IPed) N-Cad with Cx43 proteins as previously described [26], [28]. We found a greater amount of IPed Cx43 that was associated with a lesser abundance of co-IPed N-Cad (Fig. 2F). While it may not be an optimal approach to compare protein abundance detected from using two different antibodies in the setting of immunoblotting, our co-IP data further confirmed the relationship between Cx43 and N-Cad found in immunostaining images. Both results from using either IHC or co-IP techniques indicate that although N-Cad is part of the Cx43 molecular complex, the two molecules may not interact in a one-to-one matched abundance. This mismatched abundance of colocalized Cx43 and N-Cad and interior localization of N-Cad to Cx43 plaque is consistent with the findings from several other cellular systems [29]–[31].

#### 4.2. Developing a blur algorithm to identify ID co-localized Cx43_E-E_ and non-co-localized Cx43_S-S_

**Figure 3:**
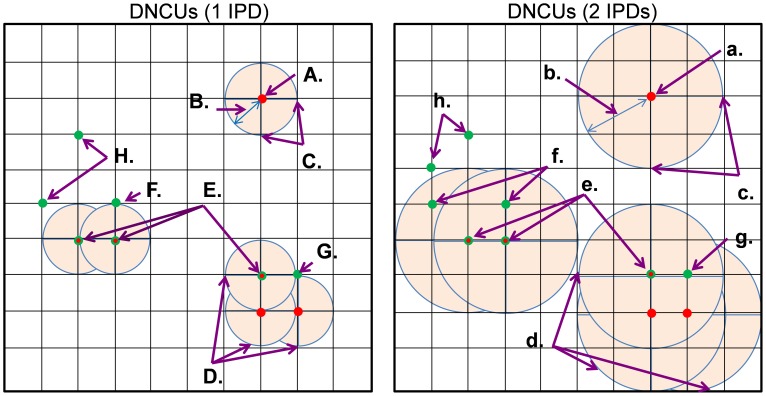
Schematic diagram of the blur algorithm with a dilated N-Cad unit (DNCU) radius of 1 inter-pixel distance (IPD) and 2 IPDs. **A&a**. An N-Cad pixel at the center of a DNCU (**C&c**). Dilation radius equals to 1 IPD and 2 IPD (**B&b**). **D&d**. 3 overlapping adjacent DCNUs (R = 1 IPD & 2 IPDs, respectively) containing 3 adjacent N-Cad pixels. **E&e**. Three Cx43 pixels that are pixel-by-pixel matched with three N-Cad pixels, respectively. **F-G & f-g**. Two Cx43 pixels that do not co-localize with any N-Cad pixel-by-pixel but are included within the area of one of DCNU. All the DNCU covered Cx43 pixels (**E-G & e-g**) are defined as Cx43_E-E_. **H&h**. Two Cx43 pixels that are located outside of all DCNU covered areas and are therefore categorized as Cx43_S-S_.

To account for the possible lack of one-to-one matched abundance, we developed a blur algorithm to identify Cx43_E-E_ signals including both N-Cad associated stellate Cx43 signals and N-Cad pixel-by-pixel matched Cx43 signals. This was achieved by using an N-Cad base area that covered the stellate Cx43 pixels. To form the base area each positive-stained N-Cad pixel (Figs. 3A, 3a) was computationally dilated at a certain radius (number of inter-pixel distance (IPD); Figs. 3B, 3b) to create a Dilated N-Cadherin Unit (DNCU; Figs. 3C, 3c) using a disk-shaped structuring element pre-coded in Matlab [32], [33]. An ID area between two adjacent myocytes could be composed of a group of adjacent DNCUs. Figs. 3A-3H are diagrams that schematically define a single DNCU and multiple DNCUs with a radius of 1 IPD, and Figs 3a-3h show diagrams of DNCUs with a radius of 2 IPDs. Cx43 pixels (green dots; Figs. 3E-3G and 3e-3g) that were covered by either a single DNCU or a group of adjacent DNCUs were considered as N-Cad co-localized Cx43_E-E_. These DNCU covered Cx43 pixels included both N-Cad pixel-by-pixel matched Cx43 (Figs. 3E,3e) and stellate Cx43 pixels (Figs. 3F-3G, 3f-3g). Cx43 pixels (Figs. 3H, 3h) located outside of the DNCUs were defined as Cx43_S-S_.

#### 4.3. Determining an optimal radius of DNCUs

**Figure 4:**
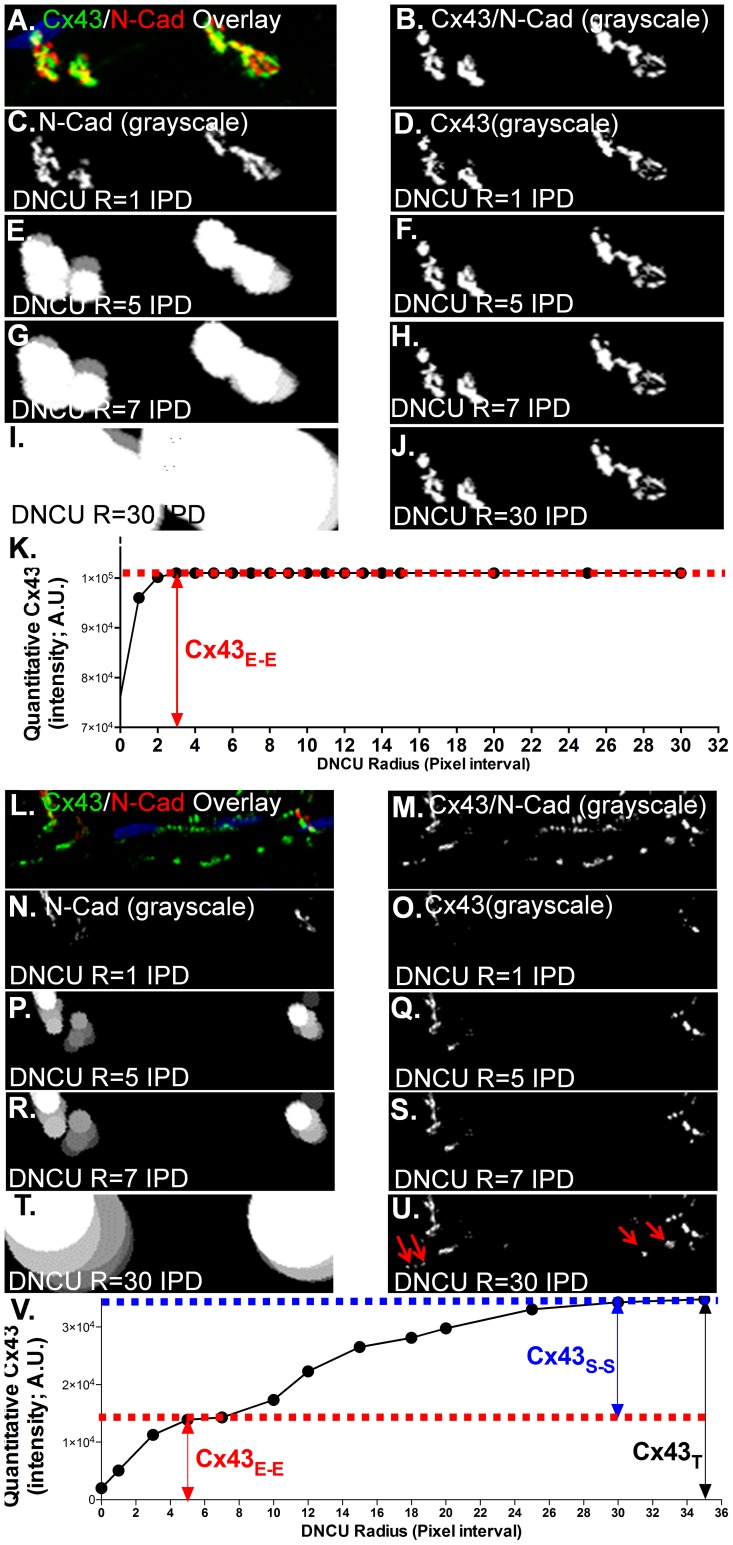
Determining an optimal radius of DNCU for Cx43_E-E_ and Cx43_S-S_ quantification. A-B & L-M. Representative Cx43 (green) and N-Cad (red) double immunostaining confocal images (**A, L**) and grayscale images (**B, M**) of enlarged single myocytes containing Cx43_E-E_ only (**A**) or both Cx43_E-E_ and Cx43_S-S_ (**L**). **C-J & N-U**. Grayscale images of the enlarged single myocytes with an incrementally increased DNCU radius (1, 5, 7, 30 IPD, respectively) for both N-Cad (left column) and Cx43 (right column). Arrows (**U**) indicate the inclusion of lateralized Cx43 by an extensively enlarged radius of the DNCU. **K & V**. Summarized data of quantitative Cx43 values corresponding to a continuously increased radii of DNCU when myocytes present Cx43_E-E_ only (**K**) or present both Cx43_E-E_ and Cx43_S-S_ (**V**). The Y-axis value of the first plateau reflects quantitative amount of ID located Cx43_E-E_ (red label) and the difference of the Y-axis value between the first and second plateaus indicates the amount of lateralized Cx43_S-S_.

We quantified all the Cx43 pixels that were covered by a group of DNCUs and analyzed the relationship between the Cx43 value and an incrementally extended DNCU radius (from 0 up to 40 IPDs; Fig 4). When all the Cx43 located at the ID areas and Cx43_S-S_ pixels were absent (Fig. 4A), all positive-stained Cx43 pixels were defined as the Cx43_E-E_ pixels. The quantitative value of Cx43 rapidly increased when the radius of DNCU was extended to 5 IPDs of the DNCU radius (Figs. 4C-4F). When the DNCU radius exceeded 5 IPDs, the quantitative value of Cx43 reached a peak and then remained at the plateau. At this plateau phase, the quantitative Cx43 peak value was not influenced by continuously increased DNCU radius (Figs. 4G-4J, 4K; red arrow). However, when both Cx43_E-E_ and Cx43_S-S_ were present (Fig. 4L), two peak plateaus of quantitative Cx43 values were found along with a continuously increased DNCU radius (up to 30 IPDs; Figs. 4N-4V). Fig. 4V shows that the value of Cx43 increased rapidly when IPD <5. When the DNCU radius increased up to 5 IPDs, the quantitative Cx43 value remained at a plateau. After the first plateau, continuously increasing the DNCU radius led to a second fast rising phase of quantitative Cx43 followed by a second plateau phase (Fig. 4V; blue arrow). While the first plateau phase reflects the amount of N-Cad associated Cx43_E-E_ (Fig. 4V, red arrow), the second plateau phase was caused by the inclusion of N-Cad non-co-localized Cx43_S-S_ signals (DNCU-uncovered Cx43; Fig. 4V, blue arrow). Thus, the Y-axis value of the first plateau phase reflects the quantitative amount of Cx43_E-E_, while the difference between the first and second plateaus at the Y-axis represents the quantitative amount of Cx43_S-S_.

### 5. Using N-Cad as an internal reference for imaging quantification

**Figure 5:**
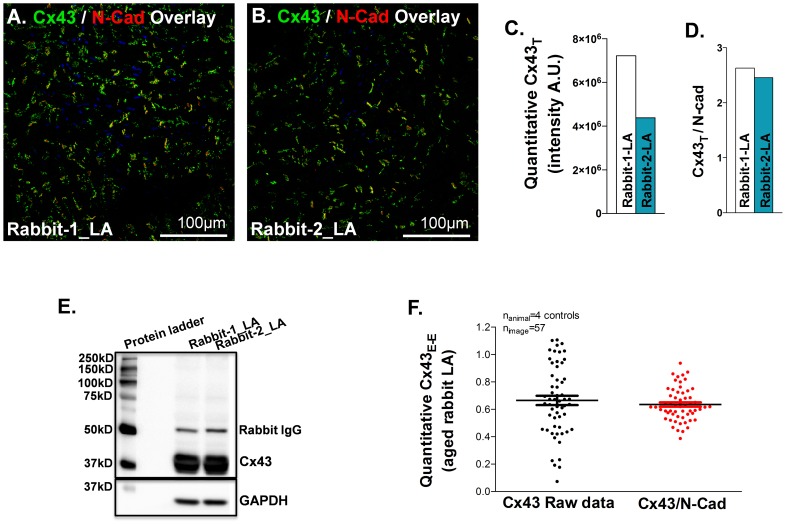
Using N-Cad as an internal housekeeping reference for imaging quantification. **A-B**. Confocal images of Cx43 (green) and N-Cad (red) double immunostaining on two LA tissue sections obtained from two young rabbits (Rabbit-1 and Rabbit-2). **C**. Difference of quantitative total Cx43 raw data between the two confocal images obtained from Rabbit-1 and Rabbit-2 LA. **D**. A comparable amount of total Cx43 between the two rabbit LA samples when Cx43 raw data was normalized to N-Cad. **E**. Immunoblotting image of total Cx43 and GAPDH protein expression in Rabbit-1 and Rabbit-2 LA tissue homogenates. **F**. Comparison of quantitative immuno-stained Cx43 signal intensity with or without N-Cad normalization in aged rabbit LA.

Tissue sectioning and histological processing variability can impact Cx43 quantification. Fig. 5 shows an example of different quantitative results of Cx43 from two healthy young rabbit hearts. One image of a Cx43/N-Cad double immunostained LA section from young healthy rabbit 1 (Rabbit-1_LA) apparently presented a greater amount of Cx43 than that of another LA section from health young rabbit 2 (rabbit-2_LA; Figs. 5A-5C). However, after normalizing quantified Cx43 raw data to the co-stained N-Cad quantitative values, abundances of total Cx43 on the two tissue sections were comparable (Fig. 5D). To further confirm the protein expression levels of Cx43 in the two healthy young rabbit LA tissue samples, an immunoblotting assay was performed. Fig. 5E reveals that the Cx43 protein expression levels were similar in the two young rabbit LA samples (Fig. 5D). Immunoblotting results were therefore consistent with the quantitative Cx43 result normalized with N-Cad. Our data suggest that the difference of quantitative IHC Cx43 values between the two young rabbit atrial sections were not due to Cx43 expression variation. To further validate the effect of N-Cad normalization in reducing quantification errors, we quantified Cx43/N-Cad double stained images from four aged rabbit atrial sections. Fig. 5F shows N-Cad normalization significantly reduced variations of quantified immunostained Cx43 signals. Thus, our results suggest that N-Cad could be an effective internal reference for eliminating quantification errors due to tissue sectioning and tissue fiber orientation variables.

### 6. Comparing quantitative Cx43 results using layer-by-layer confocal Z-stack image quantification and maximum projection image quantification

**Figure 6:**
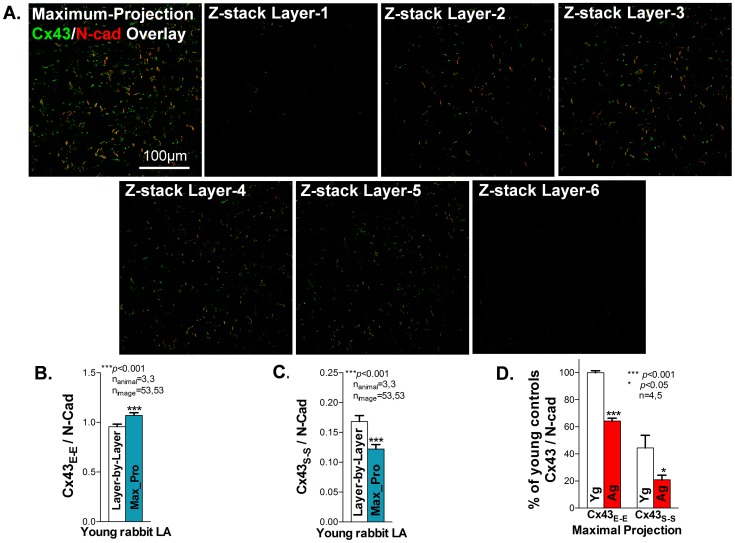
Comparing results of quantitative Cx43 distribution and abundance using layer-by-layer and maximum projection imaging quantification. **A**. Representative sequential confocal images of Cx43 (green) and N-Cad (red) double immuno-stained young rabbit LA from focal layer 1 to layer 6. **B**. Histogram of quantified Cx43 immuno-stained signals from the maximum projection image. **C-D**. Summarized data of quantified Cx43_E-E_ and Cx43_S-S_ on young rabbit LA (n = 3; 53 images of each rabbit LA section) using layer-by-layer (**B**) and maximum projection (**C**) imaging quantification approaches. **D**. Pooled data of quantified Cx43_E-E_ and Cx43_S-S_ in young and aged rabbit LA using Layer-by-Layer imaging quantification from Z-stack confocal images (n = 4, 5; *p<0.001).

A processed maximum projection confocal image from sequential Z-stack confocal images is commonly used to quantify Cx43 abundance in IHC stained sections. However, the amount of ID associated Cx43 pixels can vary in distribution and abundance at different confocal scanning layers due to changing fiber orientation in different focal planes. Fig. 6A shows apparently varied abundance of double immuno-stained Cx43 and N-Cad signals at different confocal scanning layers of Z-stack sequential confocal images obtained from a young rabbit atrial tissue section. To determine quantitative differences between using maximum projection image and layer-by-layer image processing, we processed 53 sets of Z-stack confocal images obtained from three young rabbit LA tissue sections. We first quantified Cx43_E-E_ and Cx43_S-S_ layer-by-layer on each image of a total of 6–10 sequential Z-stack confocal images. A maximum projection image processed from the same Z-stack sequential images was also quantified. Before comparing the quantitative results from using the two approaches, all the quantified Cx43 data were normalized with N-Cad as an internal normalization procedure. Fig. 6A shows the representative sequential confocal images of Cx43 (green) and N-Cad (red) double immuno-stained young rabbit LA. Fig. 6B shows that the quantitative amount of Cx43_E-E_ from the maximum projection images was higher than the sum result of using layer-by-layer quantification approach, while Cx43_S-S_ was lower than that of layer-by-layer quantification approach (Fig. 6C). These results suggest that a layer-by-layer quantification approach could minimize overestimation or underestimation of Cx43_E-E_ and Cx43_S-S_ compared to the maximum projection image quantification method.

### 7. Significantly reduced Cx43_E-E_ and Cx43_S-S_ in aged rabbit LA quantified using the blur algorithm on both layer-by-layer confocal Z-stack images and maximum projection images

We have previously reported that aged rabbit LA exhibited a reduced abundance of ID co-localized junctional Cx43 compared to that of young rabbit LA using pixel-by-pixel quantification algorithm from maximum projection confocal images [16]. Here, we re-quantified those Cx43/N-Cad double immuno-stained Z-stack confocal images obtained from 4 young and 5 aged rabbits [16] using our novel blur algorithm. We found that quantitative junctional Cx43_E-E_ in the aged rabbit LA was reduced by 36% using the blur algorithm (Fig. 6D), while the amount of Cx43_E-E_ was reduced by 25% in our previously reported result using the pixel-by-pixel algorithm (Fig. 2E of [16]). Moreover, we found that both ID co-localized Cx43_E-E_ and side-by-side distributed Cx43_S-S_ were reduced significantly in aged rabbit LA using our blur algorithm with the layer-by-layer quantification approach (Fig. 6D). The additional 11% reduction in Cx43 in the aged rabbit atrial sections from using our blur algorithm is likely due to the underestimation of stellate Cx43 in control rabbit sections from using the pixel-by-pixel method. This result was consistent with our previous finding of a critical role for reduced Cx43 abundance in atrial arrhythmia development in aged atrium.

### 8. Masson's Trichrome staining and imaging

Given the perceived importance of interstitial fibrosis as a component of cardiac arrhythmia substrate development via structural remodeling, we additionally sought to automate IC quantification through complementary image processing steps. Masson's Trichrome (MT) staining was used to detect deposited collagen on sections of atrial tissue [18], [34]. Paraffin embedded tissue sections were treated with the following steps: 1) de-waxed with xylene, and then hydrated in distilled water, 2) mordanted in Bouin's solution for 1 hour at 56°C, 3) incubated with Weigert's hematoxylin for 10 minutes, 4) put in Biebrich scarlet-acid fuchsin for 2 minutes, 5) incubated in phosphomolybdic-phosphotungstic acid solution for 10–15 minutes, 6) then put in aniline blue solution for 5 minutes, and 7) placed in 1% acetic acid solution for 3–5 minutes and dehydrated with 95% and absolute alcohol, cleared with two or three changes of xylene and then mounted with synthetic resin. For each tissue section, 15 to 20 bright field images were taken from each MT stained atrial tissue section using a Zeiss light microscope with a 40x objective oil-immersion lens.

### 9. Interstitial Collagen Quantification of MT stained atrial images

**Figure 7:**
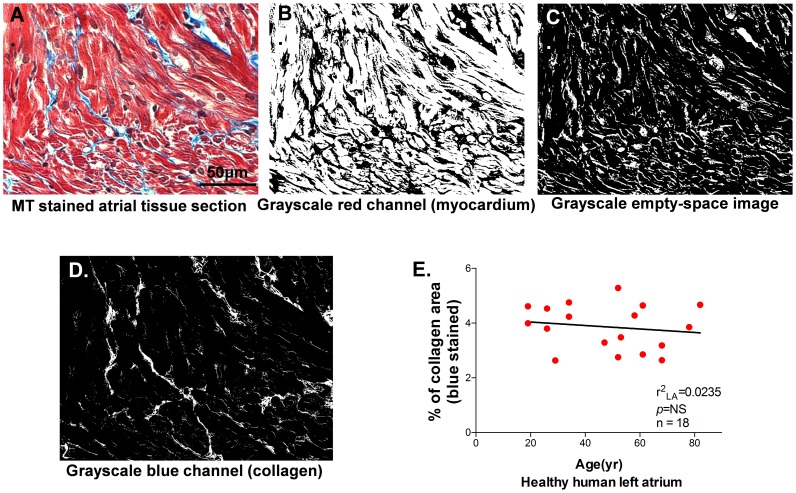
Matlab-based algorithm designed to detect interstitial collagen deposition. **A**. Representative image of Masson's Trichrome (MT) stained rabbit LA tissue (collagen  =  blue, myocardium  =  red). **B-D**. Representative grayscale images of myocardium (**B**), unstained white space (**C**), and blue stained collagen (**D**). **E.** Summarized data of quantified interstitial collagen (blue area) in healthy human LA with increasing age (n = 18).

MT stains collagen blue, myoplasm red and nuclei brown. When MT stained RGB images were converted into grayscale images, each pixel was differentiated according to the total amount of emitted light, since less light produces dark pixels and more light generates brighter pixels. Each image of a single color channel consisted of discrete pixels with various brightness intensities from 0 to 255. We therefore thresholded individual channels in each RGB image to initially segment tissue pixels from those occupying open spaces that resulted from sectioning of heterogeneous atrial muscle and to further segment tissue pixels into collagen and non-collagen groups. The intensity values of each pixel (I) in the R (red), G (green) and B (blue) channel were denoted as I_R_, I_G_ and I_B_, respectively. Thresholds were based on mean (μ) and standard deviation (σ) of pixel intensity within each image. Because unstained empty white space of the RGB images was composed of the pixels with higher intensity values on all three channels, unstained white pixels were identified as those with I_G_>μ_G_+0.9σ_G_ and I_R_>μ_R_+0.9σ_R_. Muscle/nuclei pixels had relatively high I_R_ with low I_B_ and I_G_ (I_B_<μ_B_+0.3σ_B_ and I_G_<μ_G_-0.1σ_G_). Collagen pixels had relatively high I_B_ (I_B_>μ_B_+0.9σ_B_) and low I_R_ (I_R_<μ_R_+0.25σ_R_) as well as medium I_G_ (μ_G_+0.75σ_G_<I_G_ <μ_G_+2σ_G_). Any pixels identified as being both collagen and muscle/nuclei were removed from the collagen group to avoid overestimation of IC. IC was then taken as the fraction of collagen pixels relative to total tissue pixels. Fig. 7 shows an example of the procedure with one image from an MT-stained section (A) segmented for assembly of grayscale images including tissue/nuclei (B), unstained white space (C) and collagen (D) from the original image.

In the atrium from healthy aged rabbits with normal cardiac function, we have previously discovered an unchanged interstitial collagen deposition using this quantitative algorithm. Now we quantified interstitial collagen in human left atrial appendage from the healthy donor hearts that had normal cardiac function without coexisting cardiac diseases but were not used for heart transplant for technical reasons. Fig. 7E shows that advanced age was not associated with a changed amount of interstitial collagen deposition. This result of human atrium is consistent with our previous findings in aged rabbit atrium.

## Discussion

We are the first to report this unique quantitative algorithm to quantify not only distribution but also abundance of gap junctions from cardiac tissue sections. Our main innovation involved development of a quantitative blur algorithm that used N-Cad as an internal reference to quantify both distribution and abundance of Cx43 from Cx43/N-Cad double immuno-stained cardiac tissue sections. In addition, we have developed a computer-based collagen quantification algorithm to collectively quantify interstitial collagen, excluding unstained empty areas on Masson's Trichrome stained histological slides. Our results suggest that these approaches could minimize potential overestimation or underestimation of GJ and IC in healthy and diseased hearts.

It is known that the relative amount and distribution of connexins influence electrical and chemical signal propagation throughout the heart [6], [7]. However, the importance of accurately quantifying GJ protein distribution in Cx43 immuno-stained images from diseased and aged hearts is underemphasized. For example, a reduced amount but unchanged distribution of Cx43 (the predominant connexin in ventricular GJ channels) in left ventricular epicardium was found to be associated with slowed ventricular conduction in a rapid pacing canine model of heart failure (HF), while significantly increased lateralization (enhanced redistribution) of Cx43 and slowed ventricular conduction in left ventricular epicardium was reported by others in the same canine HF model [21], [35]. Cardiac muscle fibers, especially atrial myocardial fibers, are circumferentially and longitudinally arranged in a three-dimensional myocardial space. Abundance and distribution of GJ proteins could be dramatically different when the dissected imaging focal plane of the myocardium is different. In addition, various specimen conditions such as sample-to-sample and run-to-run variations during tissue sectioning could also cause quantitative result variance. Hence, underestimation or overestimation of GJ remodeling could occur unless the confounding variables are minimized.

Cadherin is a major structure in the junctions within the intercalated discs between the two ends of two adjacent myocytes. N-Cad is a predominant cadherin molecule expressed in cardiac tissue. Studies suggest that it is an essential component in anchoring Cx43 at the ID for functional GJ formation [32], and conditional deletion of the N-Cad gene leads to ID disruption with loss of Cx43 [17]. In addition, N-Cad is also found to be unchanged in failing and aged hearts [16], [20], [21]. Thus, it is uniquely suited to serve as a housekeeper for GJ quantification. To the best of our knowledge, the published literature related to immunostained Cx43 quantification either did not include details about the method used to quantify the immunostained Cx43 or used pixel-by-pixel matched method for N-cad normalized Cx43 [36]–[38]. While valuable in its own right, such housekeeping is likely to prove beneficial as quantitative analyses mature from application in more structurally homogeneous ventricular preparations to preparations like atrial muscle in which section differences on dissected imaging focal planes are typically more pronounced. In using N-Cad as a housekeeping reference for Cx43, we emphasize that we identify a one-to-one match in which all pixels of the two types aligned with one another, but we also found abundant Cx43 pixels in close proximity in N-Cad located ID areas in both rabbit and human atria. While N-Cad mediated cell-cell adhesion is a prerequisite for Cx43 channel formation, spatial and temporal appearance and distribution between adherens junctional complex and Cx43 was discovered [2], [3], [17], [29]. In Cx43 and N-Cad double immunostained images, disproportional amounts of colocalized Cx43 and N-Cad and interior localization of N-Cad to Cx43 plaque was found in a number of cellular systems such as NIH3T3 cells, HeLa cells, and rat cardiomyocytes [29]–[31]. In this regard, we note that our Cx43 immunoprecipitation results from human atrium also revealed N-Cad associated with Cx43 as one of the components [26], [27], [34] of a Cx43 macromolecular complex. We believe the main value in anchoring pixels comprising the ID for segmentation into Cx43_E-E_ and Cx43_S-S_ types was that stellate Cx43 pixels in close proximity to N-Cad in such regions were considered as Cx43_E-E_. To complete this analysis we developed a novel blur algorithm in which optimal radii for DNCUs were quantified from differences between the first and second plateaus following iterative adjustment of IPD. Iterative adjustment was beneficial in that the first plateau provided a quantitative measure of Cx43_E-E_ while the difference between peaks provided a quantitative measure of Cx43_S-S_. Thus, we demonstrated that our novel blur algorithm was able to effectively identify the Cx43 clusters that are in close proximity in the N-cad plaque evident from the bi-phase curve of quantified Cx43 when lateralized Cx43 is present.

Consideration of N-Cad alongside Cx43 as described here provided a further benefit when used with Z-stack confocal images because it limited potential artifacts associated with the atrial muscle's heterogeneous architecture. Using maximal projection images, we found potential overestimation of Cx43_S-S_ and underestimation of Cx43_E-E_ resulting from difficulties in reconstructing ID regions in adjacent focal layers due to spatial difference in N-Cad pixel locations along with altered fiber orientations. Expanding our method to a layer-by-layer approach, we were able to confirm the previously reported result of markedly reduced junctions Cx43_E-E_ in aged rabbit LA [16]. Treating sequential images as volumes in which IDs spanned multiple layers led to our specific identification of dramatically reduced Cx43_S-S_ abundance at lateralized sites in aged rabbit LA. The results of reduced overall Cx43 abundance and unchanged Cx43 distribution pattern in aged rabbit LA were consistent with our previous findings that suppressed Cx43 abundance plays a critical role in impaired intercellular coupling and enhanced propensity for atrial arrhythmias in aged hearts [16].

In addition to GJ remodeling, interstitial fibrosis may also impact intercellular coupling by creating a barrier of dense and disorganized collagen fibrils that physically block gap junctional channels between adjacent myocytes [22]. This could cause a slowing of action potential propagation and zigzag-like transverse propagation that can ultimately lead to an increased susceptibility to arrhythmias in response to premature stimuli [18], [22]–[25]. To assess structural remodeling in aged and diseased hearts, MT staining is a unique three-color staining method that is widely used for myocardial structures. While image processing with manual outlining has been the main method used to date, use of RBG-channel based thresholding as we described here takes advantage of unique color pixel features, which are the features we considered in quantifying blue stained collagen in cardiac tissue sections. In considering our implementation, it is important to recognize and exclude unstained empty areas due to tissue section processing from the analyses. That exclusion of empty space could be an effective approach to limit histological processing-caused overestimation or underestimation of structural remodeling under a range of cardiac pathological conditions. Although this technique does not have the features to correct sample-to-sample variations, same batch MT staining according to histological standards could minimize the potential staining variations if a comparison between sections from different experimental groups will be made. Because this quantitative method is an automated program, inter-operator variation from using manual techniques could be eliminated. From using this interstitial collagen quantitative algorithm, we discovered that the amount of interstitial collagen deposition was unchanged with advanced age in human atrium from the healthy donor hearts without coexisting heart diseases. This is consistent with the result obtained from aged rabbit atrium.

Taken together, we have provided comprehensive information regarding our novel and unique quantitative algorithms for assessing cardiac gap junction distribution and abundance as well as interstitial structural remodeling. The results of using the two novel methods will significantly improve our understanding of functional consequences of molecular and structural remodeling in cardiac arrhythmia development in aged and diseased hearts.

## References

[1] Go AS, Hylek EM, Phillips KA, Chang Y, Henault LE, et al (2001) Prevalence of diagnosed atrial fibrillation in adults: national implications for rhythm management and stroke prevention: the AnTicoagulation and Risk Factors in Atrial Fibrillation (ATRIA) Study. JAMA 285: 2370–2375. 11343485 10.1001/jama.285.18.2370

[2] Giepmans BN (2004) Gap junctions and connexin-interacting proteins. Cardiovasc Res 62: 233–245. 15094344 10.1016/j.cardiores.2003.12.009

[3] Hertig CM, Butz S, Koch S, Eppenberger-Eberhardt M, Kemler R, et al (1996) N-cadherin in adult rat cardiomyocytes in culture. II. Spatio-temporal appearance of proteins involved in cell-cell contact and communication. Formation of two distinct N-cadherin/catenin complexes. J Cell Sci 109 (Pt 1): 11–20. 8834786 10.1242/jcs.109.1.11

[4] Severs NJ, Bruce AF, Dupont E, Rothery S (2008) Remodelling of gap junctions and connexin expression in diseased myocardium. Cardiovasc Res 80: 9–19. 18519446 10.1093/cvr/cvn133 PMC2533424

[5] Verheule S, van Kempen MJ, te Welscher PH, Kwak BR, Jongsma HJ (1997) Characterization of gap junction channels in adult rabbit atrial and ventricular myocardium. Circ Res 80: 673–681. 9130448 10.1161/01.res.80.5.673

[6] Bruzzone R, White TW, Paul DL (1996) Connections with connexins: the molecular basis of direct intercellular signaling. Eur J Biochem 238: 1–27. 8665925 10.1111/j.1432-1033.1996.0001q.x

[7] Saffitz JE, Davis LM, Darrow BJ, Kanter HL, Laing JG, et al (1995) The molecular basis of anisotropy: role of gap junctions. J Cardiovasc Electrophysiol 6: 498–510. 7551319 10.1111/j.1540-8167.1995.tb00423.x

[8] van der Velden HM, Ausma J, Rook MB, Hellemons AJ, van Veen TA, et al (2000) Gap junctional remodeling in relation to stabilization of atrial fibrillation in the goat. Cardiovasc Res 46: 476–486. 10912458 10.1016/s0008-6363(00)00026-2

[9] Fujimura O, Klein GJ, Yee R, Sharma AD (1990) Mode of onset of atrial fibrillation in the Wolff-Parkinson-White syndrome: how important is the accessory pathway? J Am Coll Cardiol 15: 1082–1086. 2312962 10.1016/0735-1097(90)90244-j

[10] Elvan A, Huang XD, Pressler ML, Zipes DP (1997) Radiofrequency catheter ablation of the atria eliminates pacing-induced sustained atrial fibrillation and reduces connexin 43 in dogs. Circulation 96: 1675–1685. 9315564 10.1161/01.cir.96.5.1675

[11] Tieleman RG, Crijns HJ (2000) The ‘second factor' of tachycardia-induced atrial remodeling. Cardiovasc Res 46: 364–366. 10912446 10.1016/s0008-6363(00)00067-5

[12] van der Velden HM, van Kempen MJ, Wijffels MC, van Zijverden M, Groenewegen WA, et al (1998) Altered pattern of connexin40 distribution in persistent atrial fibrillation in the goat. J Cardiovasc Electrophysiol 9: 596–607. 9654224 10.1111/j.1540-8167.1998.tb00940.x

[13] Polontchouk L, Haefliger JA, Ebelt B, Schaefer T, Stuhlmann D, et al (2001) Effects of chronic atrial fibrillation on gap junction distribution in human and rat atria. J Am Coll Cardiol 38: 883–891. 11527649 10.1016/s0735-1097(01)01443-7

[14] Kanagaratnam P, Rothery S, Patel P, Severs NJ, Peters NS (2002) Relative expression of immunolocalized connexins 40 and 43 correlates with human atrial conduction properties. J Am Coll Cardiol 39: 116–123. 11755296 10.1016/s0735-1097(01)01710-7

[15] Papageorgiou P, Monahan K, Boyle NG, Seifert MJ, Beswick P, et al (1996) Site-dependent intra-atrial conduction delay. Relationship to initiation of atrial fibrillation. Circulation 94: 384–389. 8759080 10.1161/01.cir.94.3.384

[16] Hertig C, Pye AD, Hyatt AD, Davis SS, McWilliam SM, et al (1996) Vaccinia virus-expressed bovine ephemeral fever virus G but not G(NS) glycoprotein induces neutralizing antibodies and protects against experimental infection. J Gen Virol 77 (Pt 4): 631–640. 8627251 10.1099/0022-1317-77-4-631

[17] Li J, Levin MD, Xiong Y, Petrenko N, Patel VV, et al (2008) N-cadherin haploinsufficiency affects cardiac gap junctions and arrhythmic susceptibility. J Mol Cell Cardiol 44: 597–606. 18201716 10.1016/j.yjmcc.2007.11.013 PMC2314673

[18] Platonov PG, Mitrofanova LB, Orshanskaya V, Ho SY (2011) Structural abnormalities in atrial walls are associated with presence and persistency of atrial fibrillation but not with age. J Am Coll Cardiol 58: 2225–2232. 22078429 10.1016/j.jacc.2011.05.061

[19] Severs NJ, Dupont E, Coppen SR, Halliday D, Inett E, et al (2004) Remodelling of gap junctions and connexin expression in heart disease. Biochim Biophys Acta 1662: 138–148. 15033584 10.1016/j.bbamem.2003.10.019

[20] Ai X, Zhao W, Pogwizd SM (2010) Connexin43 knockdown or overexpression modulates cell coupling in control and failing rabbit left ventricular myocytes. Cardiovasc Res 85: 751–762. 19880431 10.1093/cvr/cvp353 PMC2860702

[21] Shaner NC, Campbell RE, Steinbach PA, Giepmans BN, Palmer AE, et al (2004) Improved monomeric red, orange and yellow fluorescent proteins derived from Discosoma sp. red fluorescent protein. Nat Biotechnol 22: 1567–1572. 15558047 10.1038/nbt1037

[22] Anyukhovsky EP, Sosunov EA, Plotnikov A, Gainullin RZ, Jhang JS, et al (2002) Cellular electrophysiologic properties of old canine atria provide a substrate for arrhythmogenesis. Cardiovasc Res 54: 462–469. 12062351 10.1016/s0008-6363(02)00271-7

[23] Koura T, Hara M, Takeuchi S, Ota K, Okada Y, et al (2002) Anisotropic conduction properties in canine atria analyzed by high-resolution optical mapping: preferential direction of conduction block changes from longitudinal to transverse with increasing age. Circulation 105: 2092–2098. 11980690 10.1161/01.cir.0000015506.36371.0d

[24] Hayashi H, Wang C, Miyauchi Y, Omichi C, Pak HN, et al (2002) Aging-related increase to inducible atrial fibrillation in the rat model. J Cardiovasc Electrophysiol 13: 801–808. 12212701 10.1046/j.1540-8167.2002.00801.x

[25] Verheule S, Sato T, Everett Tt, Engle SK, Otten D, et al (2004) Increased vulnerability to atrial fibrillation in transgenic mice with selective atrial fibrosis caused by overexpression of TGF-beta1. Circ Res 94: 1458–1465. 15117823 10.1161/01.RES.0000129579.59664.9d PMC2129102

[26] Ai X, Pogwizd SM (2005) Connexin 43 downregulation and dephosphorylation in nonischemic heart failure is associated with enhanced colocalized protein phosphatase type 2A. Circ Res 96: 54–63. 15576650 10.1161/01.RES.0000152325.07495.5a

[27] Otsu N (1979) A threshold selection method from gray level histograms. IEEE Transactions on Systems, Man, and Cybernetics 9: 4.

[28] Ai X, Jiang A, Ke Y, Solaro RJ, Pogwizd SM (2011) Enhanced activation of p21-activated kinase 1 in heart failure contributes to dephosphorylation of connexin 43. Cardiovasc Res 92: 106–114. 21727092 10.1093/cvr/cvr163 PMC3172982

[29] Wei CJ, Francis R, Xu X, Lo CW (2005) Connexin43 associated with an N-cadherin-containing multiprotein complex is required for gap junction formation in NIH3T3 cells. J Biol Chem 280: 19925–19936. 15741167 10.1074/jbc.M412921200

[30] Shaw RM, Fay AJ, Puthenveedu MA, von Zastrow M, Jan YN, et al (2007) Microtubule plus-end-tracking proteins target gap junctions directly from the cell interior to adherens junctions. Cell 128: 547–560. 17289573 10.1016/j.cell.2006.12.037 PMC1955433

[31] Hertig CM, Eppenberger-Eberhardt M, Koch S, Eppenberger HM (1996) N-cadherin in adult rat cardiomyocytes in culture. I. Functional role of N-cadherin and impairment of cell-cell contact by a truncated N-cadherin mutant. J Cell Sci 109 (Pt 1): 1–10. 8834785 10.1242/jcs.109.1.1

[32] Liu IH, Chen SJ, Ku HH, Kao CL, Tsai FT, et al (2005) Comparison of the proliferation and differentiation ability between adult rat retinal stem cells and cerebral cortex-derived neural stem cells. Ophthalmologica 219: 171–176. 15947503 10.1159/000085250

[33] Chung SS, Fryxell GE, Lo CW (2005) Corporate environmental policy statements in mainland China: to what extent do they conform to ISO 14000 documentation? Environ Manage 35: 468–482. 15902447 10.1007/s00267-003-0085-3

[34] Li D, Fareh S, Leung TK, Nattel S (1999) Promotion of atrial fibrillation by heart failure in dogs: atrial remodeling of a different sort. Circulation 100: 87–95. 10393686 10.1161/01.cir.100.1.87

[35] Moolenaar WH, van Meeteren LA, Giepmans BN (2004) The ins and outs of lysophosphatidic acid signaling. Bioessays 26: 870–881. 15273989 10.1002/bies.20081

[36] Ripplinger CM, Li W, Hadley J, Chen J, Rothenberg F, et al (2007) Enhanced transmural fiber rotation and connexin 43 heterogeneity are associated with an increased upper limit of vulnerability in a transgenic rabbit model of human hypertrophic cardiomyopathy. Circ Res 101: 1049–1057. 17885214 10.1161/CIRCRESAHA.107.161240 PMC2366809

[37] Igarashi T, Finet JE, Takeuchi A, Fujino Y, Strom M, et al (2012) Connexin gene transfer preserves conduction velocity and prevents atrial fibrillation. Circulation 125: 216–225. 22158756 10.1161/CIRCULATIONAHA.111.053272 PMC3260348

[38] Remo BF, Qu J, Volpicelli FM, Giovannone S, Shin D, et al (2011) Phosphatase-resistant gap junctions inhibit pathological remodeling and prevent arrhythmias. Circ Res 108: 1459–1466. 21527737 10.1161/CIRCRESAHA.111.244046 PMC3126103

